# Developing a sampling methodology for timely reporting of population‐based COVID‐19‐associated hospitalization surveillance in the United States, COVID‐NET 2020–2021

**DOI:** 10.1111/irv.13089

**Published:** 2023-01-10

**Authors:** Alissa O'Halloran, Michael Whitaker, Kadam Patel, A. Elizabeth Allen, Kennon R. Copeland, Carrie Reed, Sue Reynolds, Christopher A. Taylor, Fiona Havers, Lindsay Kim, Kirk Wolter, Shikha Garg

**Affiliations:** ^1^ COVID‐19 Response Team Centers for Disease Control and Prevention Atlanta Georgia USA; ^2^ General Dynamics Information Technology Atlanta Georgia USA; ^3^ NORC The University of Chicago Chicago Illinois USA

**Keywords:** COVID‐19, hospitalizations, population‐based surveillance, sampling, survey methods

## Abstract

**Background:**

The COVID‐19‐Associated Hospitalization Surveillance Network (COVID‐NET) required a sampling methodology that allowed for production of timely population‐based clinical estimates to inform the ongoing US COVID‐19 pandemic response.

**Methods:**

We developed a flexible sampling approach that considered reporting delays, differential hospitalized case burden across surveillance sites, and changing geographic and demographic trends over time. We incorporated weighting methods to adjust for the probability of selection and non‐response, and to calibrate the sampled case distribution to the population distribution on demographics. We additionally developed procedures for variance estimation.

**Results:**

Between March 2020 and June 2021, 19,293 (10.4%) of all adult hospitalized cases were sampled for chart abstraction. Variance estimates for select variables of interest were within desired ranges.

**Conclusions:**

COVID‐NET's sampling methodology allowed for reporting of robust and timely, population‐based data on the clinical epidemiology of COVID‐19‐associated hospitalizations and evolving trends over time, while attempting to reduce data collection burden on surveillance sites. Such methods may provide a general framework for other surveillance systems needing to quickly and efficiently collect and disseminate data for public health action.

## 1 INTRODUCTION

The COVID‐19 pandemic has resulted in over 146 million infections and 7.5 million hospitalizations in the United States (Estimated Disease Burden of COVID‐19 | CDC).^1^, ^2^ The Centers for Disease Control and Prevention's (CDC) COVID‐19‐Associated Hospitalization Surveillance Network (COVID‐NET) was developed in March 2020 to monitor population‐based rates of COVID‐19‐associated hospitalizations and to provide timely data on clinical characteristics and outcomes of hospitalized patients with COVID‐19 to inform the US pandemic response. Due to the overwhelming burden of COVID‐19‐associated hospitalizations and the substantial time and resources required to conduct detailed medical chart abstractions, it was not feasible to quickly abstract clinical data on every case. In the initial weeks of the pandemic, clinical estimates were generated based on data available at that time, which was not systematically collected and was therefore not generalizable to COVID‐NET's surveillance area. We developed a probability‐based sampling strategy to allow for timely collection and dissemination of detailed clinical data on a representative sample of hospitalized cases on an ongoing basis throughout the pandemic. This sampling strategy would allow for continued monitoring of COVID‐19 severity among hospitalized cases throughout the pandemic.

While established survey^3^, ^4^, ^5^, ^6^ and sampling^7^, ^8^, ^9^, ^10^, ^11^ tenets would inform the sampling approach, COVID‐NET faced several unique challenges. Due to reporting delays, the sampling frame from which the sample was selected (i.e., COVID‐19‐associated hospitalizations within the catchment area) was not completely ascertained when sampled cases needed to be drawn. Therefore, the sampling methodology needed to minimize the impact of case reporting delays. Also, the 14 surveillance catchment areas varied widely in population size (ranging from a small population center of 100,000 persons to a state of six million persons), yielding varying data collection burden across sites. Shifting geographic outbreaks and changing epidemiology over the course of the pandemic also yielded varying numbers of cases over time by site and age group and meant that sampling rates would need to change over time within sampling strata.

COVID‐NET was designed using the infrastructure of the Influenza Hospitalization Surveillance Network (FluSurv‐NET), an analogous surveillance platform that first employed sampling during the 2017–2018 season.^12^, ^13^, ^14^ We aimed to devise a sampling methodology that (1) enabled timely collection and dissemination of clinical data, (2) produced robust population‐based estimates while minimizing sampling and surveillance biases, (3) allowed for adaptability given the unknown pandemic trajectory and changing data needs, and (4) included explicit analytic provisions to account for the sample design. Here, we describe the sampling methods that were successfully employed to rapidly provide epidemiologic and clinical data on COVID‐19‐associated hospitalizations throughout the first 16 months of the US pandemic.

## 2 METHODS

### 2.1 Description of the surveillance system

COVID‐NET^15^, ^16^ conducts population‐based surveillance for laboratory‐confirmed COVID‐19‐associated hospitalizations in all ages in 99 counties within 10 states participating in the long‐standing Emerging Infections Program (CA, CO, CT, GA, MD, MN, NY, NM, OR, and TN) and four states participating in the Influenza Hospitalization Surveillance Project (IA, MI, OH, and UT). COVID‐NET covers a catchment population of approximately 32 million persons (almost 10% of the US population). Surveillance staff systematically review hospital, laboratory, and notifiable disease databases to identify all COVID‐19‐associated hospitalizations among catchment area residents. Hospitalized patients who are catchment area residents and have a positive SARS‐CoV‐2 test during hospitalization or within 14 days before admission are included.

### 2.2 Estimating population‐based COVID‐19‐associated hospitalization rates

**FIGURE 1:**
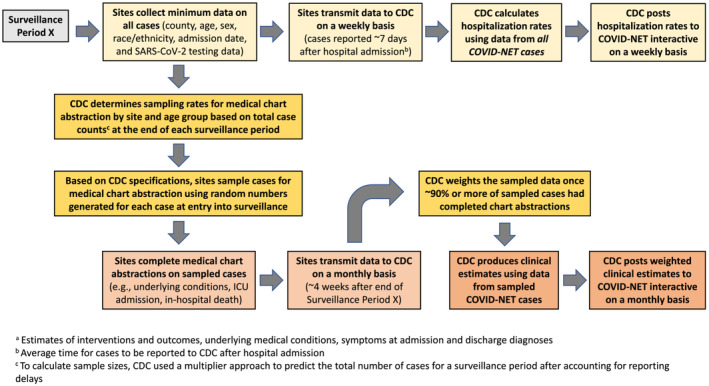
Data workflow and timelines for COVID‐NET sampling and public reporting of rate data and clinical estimates^a^

Surveillance sites transmit a minimum dataset (county, age, sex, race/ethnicity, hospital admission date, and SARS‐CoV‐2 testing data) on all identified cases to CDC on a weekly basis to produce COVID‐19‐associated hospitalization rates stratified by key demographics. Incidence rates are calculated using COVID‐NET cases as the numerator and the National Center for Health Statistics' Vintage bridged‐race postcensal population estimates^17^ as the denominator. Rates are posted weekly to CDC's COVID‐NET interactive^18^ and COVID Data Tracker^2^ webpages. On average, minimum data for each case are provided to CDC within 7 days of hospital admission (Figure 1); however, reporting delays vary by surveillance site and epidemiologic week and can range from 1 day to several weeks. By 6 weeks from admission, >90% of cases have been reported.

### 2.3 Characteristics of hospitalized COVID‐NET cases

Using a standardized case report form (CRF) with >400 variables, trained surveillance staff conduct in‐depth medical chart abstractions on sampled cases, including data on underlying medical conditions, intensive care unit (ICU) admission, mechanical ventilation, receipt of COVID‐19 vaccination or treatment, and death during hospitalization. Data on the prevalence of these clinical characteristics and outcomes are posted to COVID‐NET's interactive web pages^19^ but updated less frequently than hospitalization rates due to the substantial time needed to collect these data (Figure 1); prior to the sampling strategy implementation, the delay ranged from weeks to months.

### 2.4 General sampling approach

Because site, age, and hospital admission date were known for all cases upon first report to CDC, these variables were used to stratify cases for sampling. Sampling periods were selected because sampling needed to occur on an ongoing basis. For each sampling period, sample sizes were calculated for the entire surveillance network to achieve desired precision around clinical estimates of interest; samples were then distributed across the 14 surveillance sites. Sites drew random samples of cases based on CDC specifications, conducted medical chart abstractions on sampled cases, and transmitted data to CDC. CDC weighted the data to reflect the sample design and reported weighted clinical estimates for each period on its interactive website (Figure 1). The following sections describe these steps in detail.

#### 2.4.1 Sampling by age group

Because different age groups experienced different rates of COVID‐19‐associated hospitalization,^2^, ^18^ COVID‐NET stratified samples by age group (0–17, 18–49, 50–64, and ≥65 years). Doing so allowed for age groups to be sampled at rates inversely related to the age group's hospitalization burden. Sampling rates were adjusted over time as age groups experienced changing hospitalization rates. Because COVID‐19‐associated hospitalizations were less frequent in children, all cases <18 years were sampled; thus, the remaining sampling discussion focuses on adults.

#### 2.4.2 Sampling by period

**FIGURE 2:**
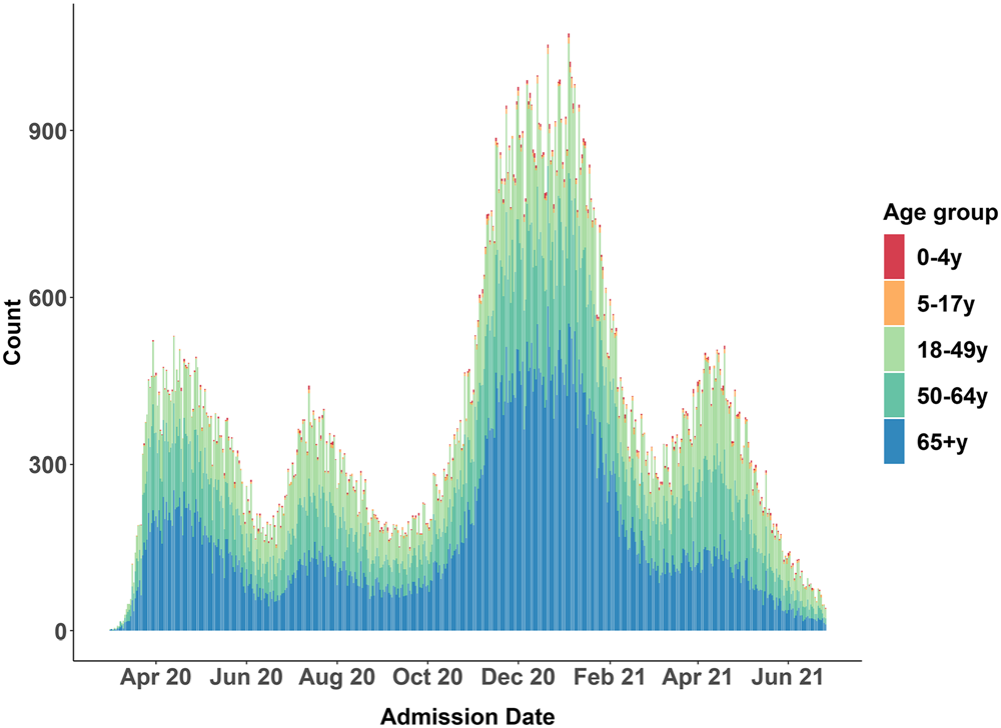
COVID‐19‐associated hospitalization counts by admission date and age group, COVID‐NET March 2020 to June 2021

In the initial months of the pandemic, sampling periods were established retrospectively based on hospitalization rate patterns. The first hospitalization wave occurred during March through May 2020 (Figure 2); thus, the first sampling period included cases hospitalized during this period. A second hospitalization wave occurred during July through August 2020; thus, the second sampling period encompassed June through September 2020. Sampling rates were not determined until after the sampling period had ended, when the number of cases had been determined. Choosing longer periods over which to sample resulted in sites waiting longer to draw samples and led to substantial delays in availability of clinical data. Starting in October 2020, the sampling period shifted to a monthly pattern, allowing sites to more quickly sample and conduct chart abstractions. Monthly sampling also allowed CDC to rapidly adjust sampling rates in response to changes in hospitalization rates.

#### 2.4.3 Sampling by surveillance site

While each COVID‐NET site followed standardized protocols for case ascertainment and data collection, case‐finding and data acquisition methods varied across sites and were governed by the site's underlying public health infrastructure and resources. Each site's catchment size also varied substantially. Therefore, sampling was stratified by site. Sites with smaller population catchment areas had fewer cases and were able to accommodate greater sampling rates, while sites with large catchment areas and more hospitalizations required smaller sampling rates. Sites also experienced surges of cases at differing times, and sampling by site allowed for varying sampling rates in response to geographic trends over time.^18^

#### 2.4.4 Sample size calculations and sampling rates

Sample size calculations were developed in the Spring of 2020 and relied on early point estimates of variables of interest (e.g., ICU admission, mechanical ventilation, and in‐hospital death) with a prevalence of approximately ≥10%. Sample sizes for all sites combined were calculated assuming simple random sampling (SRS) with the aim of producing reliable point estimates (relative standard error [RSE] or coefficient of variation [CV] < 0.3) and 95% confidence interval half‐widths <10.^20^, ^21^ Sample size calculations demonstrated that across the network, a minimum of 100 cases was necessary within each period and stratum. In practice, 100 cases were found to be insufficient because sites were not able to access patient charts for all the sampled cases. Additionally, we anticipated an increase in variance, above the level given by SRS, as a result of disproportionate allocation of the total sample size to strata and any non‐response. As such, we adjusted the required sample size to at least 200 cases per stratum across the network. We also summarized the increase in variance by the weighting effect (WEFF). The formula for *WEFF* is shown below, where *CV* is the coefficient of variation, *W*_*i*_ is the final weight for the *i*th case, and *n* is the sample size, for each period.^22^ In November 2020, weighting effects were calculated using historical data from March through October 2020, and the resulting weighting effects were used to inflate November 2020 sample sizes calculated under SRS (with the weighting effect, ~350 sampled cases were required across all sites rather than 200). After November 2020, weighting effects were considered, though not strictly applied in order to minimize burden on sites, when determining sample sizes.

$$\text{WEFF}=1+CV^{2}=1+\frac{\left(1/n\right)\sum {\left(W_{i}-\bar{W}\right)}^{2}}{{\bar{W}}^{2}}=\frac{n\sum W_{i}^{2}}{{\left(\sum W_{i}\right)}^{2}}.$$

Because at least 200–350 cases per strata were needed across all sites combined, we next established methods to produce site‐specific sampling rates. While determining these sampling rates, the following objectives were considered:

1. to achieve similar sampling rates across sites within an age group;
2. to maintain flexibility to allow over‐burdened sites to sample at smaller rates; and
3. to sample a minimum number of cases (>15) from each site, if possible (if sites had <15 cases in a given strata, 100% sampling was required).

The sampling design is further detailed in Table 1.

**TABLE 1:** Sampling rates by sampling period and age group, COVID‐NET,^a^ March 2020 to June 2021

Sampling period	18–49 years		50–64 years		65+ years	
	Total cases	Sampling rate^b^ for all sites (range)	Total cases	Sampling rate^b^ for all sites (range)	Total cases	Sampling rate^b^ for all sites (range)
March–May 2020	7390	44% (10% to 100%)	8459	13% (10% to 20%)	12,867	10% (10% to 10%)
June–September 2020^c^	10,136	20% (5% to 40%)	8413	12% (5% to 20%)	11,109	8% (5% to 10%)
October 2020	2532	11% (4% to 69%)	2677	12% (3% to 93%)	4373	7% (3% to 38%)
November 2020	4965	10% (10% to 10%)	5918	10% (10% to 10%)	11,294	5% (3% to 5%)
December 2020	5778	5% (3% to 5%)	7125	4% (3% to 5%)	14,615	2% (1% to 3%)
January 2021	5227	6% (3% to 10%)	6441	6% (3% to 10%)	12,429	3% (1% to 5%)
February 2021	2760	12% (7% to 50%)	3030	11% (7% to 50%)	5183	7% (3% to 50%)
March 2021	3057	12% (5% to 50%)	3302	11% (3% to 50%)	3749	8% (3% to 50%)
April 2021	4697	7% (2% to 70%)	4362	8% (2% to 70%)	3961	9% (2% to 70%)
May 2021	2868	17% (7% to 100%)	2270	17% (10% to 100%)	2249	19% (10% to 100%)
June 2021	1178	31% (25% to 100%)	785	44% (35% to 100%)	850	46% (35% to 100%)

^a^ COVID‐NET includes 14 US states (CA, CO, CT, IA, GA, MD, MI, MN, NM, NY, OH, OR, TN, and UT).

^b^ Actual sampling rates may have deviated slightly due to random number selection.

^c^ All sites sampled persons aged 18–29 years at 100% except CA, GA, and MD who sampled at a rate of 10%.

#### 2.4.5 Drawing samples

The sampling approach necessitated waiting until the end of each sampling period, when most cases had been ascertained. Ideally, sampling rates for a specific surveillance period would be determined very soon after a period ended. However, due to reporting delays, it took up to 6 weeks after the end of a surveillance period for CDC to obtain final case counts; case counts for the most recent weeks of a period were particularly underestimated. To address this, CDC used a multiplier method developed in‐house^23^ to predict the total number of cases by site and age group within a given period, after accounting for historical reporting delays. Doing so allowed for sampling rates to be determined 1–2 weeks after the period ended (Figure 1).

After sampling rates were determined for each period, based on CDC specifications, sites drew samples using auto‐generated random numbers (1–100) that were prospectively assigned to each new case upon entry into the surveillance database. For example, if a site needed a 10% sample, cases assigned to random numbers yielding a 10% sample were drawn. While sites were required to abstract data on a minimum number of sampled cases, they could elect to conduct chart abstractions on all cases within specified strata to allow them more power for site‐specific analyses. Because data on all cases are known for these strata, these strata are referred to as “certainty strata.”

#### 2.4.6 Data collection and reporting

Sites completed chart abstractions with a goal of submitting data for all sampled cases within 3–4 weeks after each sample draw. Sites entered sampled case data into a standardized database and transmitted data weekly to CDC. Once a majority of sites completed at least 90% of chart abstractions for sampled cases for a given period, combined data for those sites were weighted and posted to CDC's interactive webpage.^19^ Monthly clinical estimates were posted about 4–6 weeks after the end of each surveillance period, and cumulative estimates were also updated at this time (Figure 1).

### 2.5 Weighting process and variance estimation

Race/ethnicity and sex, used in the weighting process, were missing on 2% and 0.1% of sampled cases, respectively. Both fields were imputed using hot deck (single) imputation.^24^ Clinical data were weighted to reflect the probability of selection, adjusted for non‐response (CRF data completed for <100% of sampled cases), and raked to align the weighted case distribution in COVID‐NET by site, age, sex, and race/ethnicity with the COVID‐NET catchment population totals using methods previously described.^25^ Because weight adjustments yielded more dispersed weights, weights were trimmed^25^ to minimize overdispersion. The full weighting process is outlined in Table S1. After final sampling weights were created, Jackknife replicate weights^11^ were created for variance estimation. A method that limited the number of Jackknife replicates was utilized to facilitate efficient computations and is described in Text S1.

### 2.6 Evaluating the impact of the sampling scheme

To evaluate the impact of the sampling methods employed, site response rates (number of sampled cases with completed charts divided by the total number of sampled cases) were tracked over time. Weighting effects were calculated by sampling period, and observations were made on how the sampling rates, sample sizes, and response rates impacted weighting effects over time. Select point estimates, RSEs, and 95% CIs were calculated for each period for the 14 COVID‐NET states, where the variance estimation method which calculates variance from all strata was used. Observations were also made on the number of estimates exceeding the RSE cutoff of 0.3 and the CI half‐width cutoff of 10 for each period.

This activity was reviewed by CDC and was conducted consistent with applicable federal law and CDC policy (see, e.g., 45 C.F.R. part 46.102(l)(2), 21 C.F.R. part 56; 42 U.S.C. §241(d); 5 U.S.C. §552a; 44 U.S.C. §3501 et seq.). Sites participating in COVID‐NET obtained approval from their respective state and local Institutional Review Boards, as applicable.

## 3 RESULTS

Between March 1, 2020, and June 30, 2021, 186,049 adult cases with COVID‐19‐associated hospitalizations were reported to COVID‐NET, with 19,293 (10.4%) randomly sampled for medical chart abstraction. Across sampling periods and age groups, average sampling rates ranged from 5% to 44% among cases aged 18–49 years, 4% to 44% among those aged 50–64 years, and 2% to 46% among those aged ≥65 years (Table 1). Sampling rates stratified by site, age group, and time period are displayed in Table S2 and ranged from 1% to 100%. After applying final sample weights, COVID‐NET sample estimates were similar by age, sex, race/ethnicity, and site to all identified COVID‐NET cases (Table S3).

**FIGURE 3:**
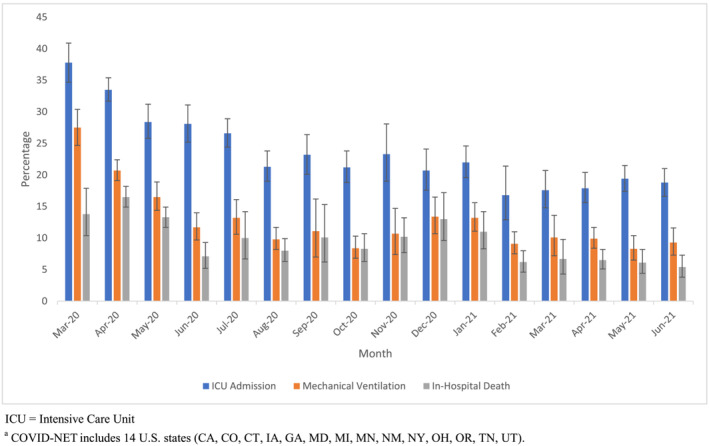
Weighted percentages and confidence intervals for select clinical interventions and outcomes by month among sampled cases, COVID‐NET,^a^ March 2020 to June 2021

Figure 3 shows the weighted proportions and 95% confidence intervals of sampled cases who required ICU admission, mechanical ventilation, and who died in‐hospital by month for the 14 COVID‐NET states; Table S4 and Figure S1 show these results by age group. All point estimates with prevalence ≥10% had RSEs < 0.3, while only a few point estimates with prevalence <10% had an RSE > 0.3. The 95% confidence interval half‐widths for all point estimates with the exception of one were <10% (Table S4). Figure 3 also demonstrates the utility of these sampled data and shows the trends in clinical estimates and outcomes over time. For example, among all ages ICU admission declined from 37.8% in March 2020 to as low as 16.8% in February 2021. In‐hospital deaths decreased during the initial months of the pandemic from a high of 16.5% in April 2020 to as low as 7.1% in June 2020 but increased to 13% in December of 2020 (Figure 3).

Final sample weights differed by site, age group, and month. Table S5 shows the sample sizes, range of weights across the network, and weighting effect by month and age group. Weighting effects ranged from 1.0 to 3.0 with a median of 1.4. The highest weighting effects were seen in the 18–49 age group during the summer months of 2020, which correlated with lower case counts and a relatively wide range of weights.

## 4 DISCUSSION

With the demand for timely surveillance data to inform the ongoing COVID‐19 pandemic response, COVID‐NET rapidly adapted methods to collect and disseminate data to meet the evolving needs of the COVID‐19 response while still maintaining data quality and integrity. This surveillance system achieved a flexible, timely sampling approach that produced statistically robust, population‐based clinical estimates down to the monthly level, while attempting to reduce data collection burden on surveillance sites. This approach has been implemented since March 2020 and has allowed COVID‐NET sites to sample as few as 1% of cases within certain strata. Rigorous evaluation of the sampling approach has demonstrated that the demographic distributions of the sample were similar to all hospitalized cases, and variance estimates for key clinical variables of interest were generally within the desired a priori ranges. The sampling approach has enabled COVID‐NET to monitor important clinical and public health parameters related to COVID‐19, including trends in clinical disease severity^19^, ^26^ and population impact of public health interventions^27^ on rates of COVID‐19‐associated hospitalization over time.

Decisions about sampling period durations and timing of sample draws were carefully considered with the goals of providing timely data and allowing for flexibility as the pandemic evolved. Ultimately, sampling in shorter periods and drawing samples soon after the surveillance period ended optimized the production of timely estimates. Modeling final case counts while accounting for reporting delays aided with timeliness. Samples were randomly drawn by sites at the time of case entry into the surveillance database, allowing sites some flexibility in managing workload. Creating a process to weight data, adjust for non‐response, and post data when approximately 90% of cases were complete for a given surveillance period further enhanced timeliness. This process allowed for estimation of monthly trends in disease severity^19^, ^26^ and timely descriptions of racial and ethnic disparities in rates of severe COVID‐19.^28^, ^29^

Sample size determinations accounted for surveillance priorities and site resources and capacity. Selecting larger sample sizes and using similar sampling rates across all sites yielded higher precision. Additionally, applying weighting effects to sample size calculations helped mitigate potential increases in variance due to differential sampling rates across sites, non‐response adjustments, or raking.^22^, ^30^, ^31^ However, during periods when case counts were unusually high and case burden was unequally distributed across sites, sampling at uniformly high rates was not feasible. For COVID‐NET, it was preferred to have complete data on fewer representative cases than to have less complete, less representative data on a larger sample. When sample size requirements were lower, and differential sampling rates were applied across sites based on case burden, non‐response was greatly reduced.

There are several limitations to COVID‐NET surveillance. COVID‐NET is a resource intensive surveillance platform which requires surveillance staff to ascertain cases and manually conduct medical chart abstractions using standardized protocols. While this approach yields accurate and consistent data across the platform, these high‐quality data come at a potential cost; clinical data are available on a monthly rather than daily or weekly basis. COVID‐NET data are also not nationally representative given that these resource intensive methods could not easily be implemented across all states; however, defined catchment areas allow for production of population‐based estimates of the COVID‐NET catchment areas rather than estimates generated from convenience samples. There were also several limitations related to the sampling approach. Reporting delays were modeled to allow for more timely sampling, but modeled sample sizes could be over or underestimated. Non‐response adjustment and raking processes also required subjective decision‐making considering surveillance goals and feasibility. While adjustments could be made on data with response rates much lower than 90%, doing so yielded a larger non‐response adjustment factor that adversely impacted variance. Finally, the sampling guidance evolved rapidly as CDC learned and adapted to challenges, which yielded a process that was inconsistent at times.

With implementation of this flexible and adaptive sampling approach, COVID‐NET has been instrumental in providing robust and timely, population‐based data on the clinical epidemiology of COVID‐19‐associated hospitalizations and evolving trends over the course of the pandemic; these data have been used to inform vaccine recommendations and other policy decisions.^16^, ^26^, ^27^, ^28^, ^29^, ^32^, ^33^, ^34^, ^35^ Such methods may provide a general framework for other surveillance systems needing to quickly and efficiently collect and disseminate large amounts of data.

## CONFLICT OF INTEREST

Kirk Wolter and Kennon A. Copeland disclosed personal fees from the Centers for Disease Control and Prevention as payment through a T&M contract.

## AUTHOR CONTRIBUTIONS

All authors contributed to the study conception and design. Material preparation, data collection, and analysis were performed by Michael Whitaker, Kadam Patel, and Alissa O'Halloran. The first draft of the manuscript was written by Alissa O'Halloran and Michael Whitaker, and all authors commented on previous versions of the manuscript. All authors read and approved the final manuscript.

## DISCLAIMER

The findings and conclusions in this report are those of the author(s) and do not necessarily represent the official position of the Centers for Disease Control and Prevention (CDC).

### PEER REVIEW

The peer review history for this article is available at https://publons.com/publon/10.1111/irv.13089.

## Supporting information


**Text S1.** Supporting Information

**Figure S1a.** Weighted percentages and confidence intervals for select clinical interventions and outcomes by month among sampled cases 18‐49 years, COVID‐NET^a^, March 2020 – June 2021
**Figure S1b.** Weighted percentages and confidence intervals for select clinical interventions and outcomes by month among sampled cases 50‐64 years, COVID‐NET^a^, March 2020 – June 2021
**Figure S1c.** Weighted percentages and confidence intervals for select clinical interventions and outcomes by month among sampled cases ≥65 years, COVID‐NET^a^, March 2020 – June 2021

**Table S1.** COVID‐NET sample weighting process
**Table S2A.** Overall sampling rates by site, surveillance period and age group 18‐49 years, COVID‐NET March 2020 – June 2021
**Table S2B.** Overall sampling rates by site, surveillance period and age group 50‐64 years, COVID‐NET March 2020 – June 2021
**Table S2C.** Overall sampling rates by site, surveillance period and age group ≥65 years, COVID‐NET March 2020 – June 2021
**Table S3.** Comparison of the distribution of demographic characteristics among all hospitalized cases versus weighted sampled hospitalized cases, COVID‐NET, March 2020 – June 2021
**Table S4.** Weighted percentages and confidence intervals for select clinical interventions and outcomes by month and age group among sampled hospitalized cases, COVID‐NET^a^, March 2020 – June 2021
**Table S5.** COVID‐NET weighting summary by surveillance period and age group, March 2020 – June 2021

## Data Availability

Data are not publicly available. Please contact corresponding author Alissa O'Halloran (idg3@cdc.gov) with data‐related questions.
