# Supplementary material for: Developing a sampling methodology for timely reporting of population‐based COVID‐19‐associated hospitalization surveillance in the United States, COVID‐NET 2020–2021
Source: Influenza Other Respir Viruses. 2023 Jan 10;17(1):e13089. doi: 10.1111/irv.13089 (PMC9835436; doi:10.1111/irv.13089)
Supplement: Supplementary file 1 — Text S1. Supporting Information [file IRV-17-e13089-s001.docx]

**S1 Text: COVID-NET Jackknife Variance Estimation**

Cases were assigned into $H$ = 45 variance strata, $h$, based on site and month, with the goal of balancing each stratum on age and race/ethnicity. Two replicate groups ($\beta$ = 1,2) were formed within each variance stratum by randomly assigning half the sample cases within the variance stratum to each replicate group, and one variance-stratum/replicate group was dropped at a time. Final weights were adjusted to account for the dropped replicate group; after dropping out variance-stratum/replicate-group $h\beta$, the corresponding replicate weights were defined as follows:

$$W_{(h\beta)h^{'}i}=\left\{ \begin{matrix} W_{h'i} & , \text{ if} h'\text{ }\neq h \\ 0 & , \text{ if }h'=h \text{and }i\in\beta\\ \frac{2}{2-1}W_{h'i} & , \text{if }h'=h \text{and }i\notin\beta, \end{matrix} \right.$$

for $h^{'}=1,\ldots,H$ and all completed cases $i$ in the sample in the variance stratum.

Let $\theta$ be a parameter of interest to be estimated and let $\hat{\theta}$ be its estimator based on the full sample and the corresponding weights. A new estimate, $\hat{\theta}_{(h\beta)}$ , was created after dropping the $h\beta$ replicate group and using the corresponding jackknife replicate weights, and the squared difference between the new estimate and the full-sample estimate was calculated. The sum of the squared differences, appropriately scaled, was the variance estimate, as follows:

$$v\left( \hat{\theta} \right)=\sum_{h=1}^{H} \left( \frac{2-1}{2} \right)\sum_{\beta=1}^{2} \left( \hat{\theta}_{\left( h\beta\right)}-\hat{\theta} \right)^{2}.$$

Another similar Jackknife replicate weight method was also developed to zero out the estimated sampling variance in certainty strata. The variance estimation approach used largely depends on the questions being addressed by the data and whether the data are being used for descriptive purposes such as surveillance or to address specific analytic questions. If the data are being used only to make inferences within the COVID-NET catchment area, the method that zeros out variance from certainty strata might be more appropriate, though it produces smaller variance estimates. COVID-NET has employed both approaches to variance estimation.
