# Supplementary material for: Developing a sampling methodology for timely reporting of population‐based COVID‐19‐associated hospitalization surveillance in the United States, COVID‐NET 2020–2021
Source: Influenza Other Respir Viruses. 2023 Jan 10;17(1):e13089. doi: 10.1111/irv.13089 (PMC9835436; doi:10.1111/irv.13089)
Supplement: Supplementary file 3 — Table S1. COVID‐NET sample weighting process Table S2A. Overall sampling rates by site, surveillance period and age group 18‐49 years, COVID‐NET March 2020 – June 2021 Table S2B. Overall sampling rates by site, surveillance period and age group 50‐64 years, COVID‐NET March 2020 – June 2021 Table S2C. Overall sampling rates by site, surveillance period and age group ≥65 years, COVID‐NET March 2020 – June 2021 Table S3. Comparison of the distribution of demographic characteristics among all hospitalized cases versus weighted sampled hospitalized cases, COVID‐NET, March 2020 – June 2021 Table S4. Weighted percentages and confidence intervals for select clinical interventions and outcomes by month and age group among sampled hospitalized cases, COVID‐NETa, March 2020 – June 2021 Table S5. COVID‐NET weighting summary by surveillance period and age group, March 2020 – June 2021 [file IRV-17-e13089-s002.docx]

**S1 Table. COVID-NET sample weighting process**

| **Step** | **Process** | **Description** |
| --- | --- | --- |
| **1** | **Imputation** | - Less than 3% of data on race/ethnicity and sex were imputed using hot deck (single) imputation where site, age group and month of admission were used to select donors |
| **2** | **Base weight** | - The base weight was calculated as the total number of COVID-NET cases divided by the number of sampled cases in each sampling period by site and age group |
| **3** | **Non-response adjustment** | - Non-response adjustment factors were calculated as the total number of sampled cases divided by the number of completed sampled cases and were stratified by site and age group - The non-response adjustment weight was the product of the base weight and the non-response adjustment factor |
| **4** | **First raking** | - A SAS macro was used to rake the non-response adjustment weights to known population totals - Four raking dimensions were used, listed in order: site by month, race/ethnicity by month, sex by month, age group by month |
| **5** | **Weight trimming** | - Weight trimming was applied to the raked weights, where $W_{i}$ is the final weight for the $i$-th case, $m$ is the median and $\sigma$ is the interquartile range of the weights.   $W_{i}=\left\{ \begin{matrix} m+2.9\sigma& \text{, if }W_{i}\geq m+3\sigma\\ max\left( 1,m-3\sigma_{F} \right) & \text{, if }W_{i}\leq max\left( 1,m-3\sigma\right) \\ W_{i} & , otherwise \end{matrix} \right.$ |
| **6** | **Second raking** | - A second round of raking was applied to adjust the trimmed weights to sum to the population totals, using the same dimensions above. - The final sample weight was obtained after iterating these dimension to convergence. |

**Supplemental Table 2A. Overall sampling rates by site, surveillance period and age group 18-49 years, COVID-NET March 2020 – June 2021**

| **Surveillance Period** | **Sampling Rate by Site For 18-49 Year Age Group** | | | | | | | | | | | | | |
| --- | --- | --- | --- | --- | --- | --- | --- | --- | --- | --- | --- | --- | --- | --- |
|  | **CA** | **CO** | **CT** | **GA** | **IA** | **MD** | **MI** | **MN** | **NM** | **NY** | **OH** | **OR** | **TN** | **UT** |
| Mar-May 2020 | 100% | 10% | 100% | 10% | 100% | 10% | 100% | 100% | 100% | 100% | 100% | 100% | 100% | 100% |
| Jun-Sept 2020^a^ | 10% | 40% | 40%^b^ | 5% | 40% | 5% | 40% | 40% ^b^ | 40% | 40% | 40% | 40% | 40% | 40% ^b^ |
| Oct 2020 | 14% | 5% | 48% | 6% | 69% | 4% | 41% | 7% | 11% | 26% | 25% | 22% | 13% | 14% |
| Nov 2020 | 10% | 10% | 10% | 10% | 10% | 10% | 10% | 10% | 10% | 10% | 10% | 10% | 10% | 10% ^b^ |
| Dec 2020 | 3% | 5% | 5% | 3% | 5% | 3% | 5% | 5% | 5% | 5% | 5% | 5% | 5% | 5% ^b^ |
| Jan 2021 | 3% | 10% | 10% | 3% | 10% | 3% | 10% | 10% | 10% | 10% | 10% | 10% | 10% | 10% |
| Feb 2021 | 7% | 15% | 15% | 7% | 50% | 7% | 30% | 15% | 15% | 15% | 30% | 30% | 15% | 30% |
| Mar 2021 | 15% | 15% | 15% | 5% | 50% | 5% | 15% | 15% | 15% | 15% | 30% | 30% | 15% | 30% |
| Apr2021 | 7% | 6% | 7% | 6% | 70% | 2% | 6% | 6% | 30% | 7% | 30% | 30% | 7% | 30% |
| May 2021 | 30% | 7% | 30% | 7% | 100% | 7% | 30% | 7% | 30% | 30% | 30% | 30% | 30% | 30% |
| Jun 2021 | 25% | 25% | 50% | 25% | 100% | 25% | 50% | 25% | 25% | 50% | 100% | 25% | 50% | 50% |

^a^ Sites at 40% sampling did 100% sampling for cases 18-29 years and 20% of cases 30-49 years yielding roughly 40% of all cases in the 18-49 age group

^b^ Site elected to do 100% of charts for this stratum

**Supplemental Table 2B. Overall sampling rates by site, surveillance period and age group 50-64 years, COVID-NET March 2020 – June 2021**

| **Surveillance Period** | **Sampling Rate by Site For 50-64 Year Age Group** | | | | | | | | | | | | | |
| --- | --- | --- | --- | --- | --- | --- | --- | --- | --- | --- | --- | --- | --- | --- |
|  | **CA** | **CO** | **CT** | **GA** | **IA** | **MD** | **MI** | **MN** | **NM** | **NY** | **OH** | **OR** | **TN** | **UT** |
| Mar-May 2020 | 20% | 10% | 20% | 10% | 20% | 10% | 20% | 20% ^a^ | 20% | 20% | 20% | 20% ^a^ | 20% ^a^ | 20% ^a^ |
| Jun-Sept 2020 | 10% | 20% | 20% | 5% | 20% | 5% | 20% | 20% ^a^ | 20% | 20% | 20% | 20% | 20% | 20% ^a^ |
| Oct 2020 | 16% | 5% | 29% | 7% | 93% | 3% | 30% | 7% | 12% | 55% | 26% | 29% | 10% | 13% |
| Nov 2020 | 10% | 10% | 10% | 10% | 10% | 10% | 10% | 10% | 10% | 10% | 10% | 10% | 10% | 10% ^a^ |
| Dec 2020 | 3% | 5% | 5% | 3% | 5% | 3% | 5% | 5% | 5% | 5% | 5% | 5% | 5% | 5% ^a^ |
| Jan 2021 | 3% | 10% | 10% | 3% | 10% | 3% | 10% | 10% | 10% | 10% | 10% | 10% | 10% | 10% |
| Feb 2021 | 7% | 15% | 15% | 7% | 50% | 7% | 30% | 15% | 15% | 15% | 30% | 30% | 15% | 30% |
| Mar 2021 | 15% | 15% | 15% | 3% | 50% | 3% | 15% | 15% | 15% | 15% | 30% | 30% | 15% | 30% |
| Apr2021 | 15% | 6% | 15% | 6% | 70% | 2% | 6% | 6% | 30% | 15% | 30% | 30% | 15% | 30% |
| May 2021 | 30% | 10% | 30% | 10% | 100% | 10% | 30% | 10% | 30% | 30% | 30% | 30% | 30% | 30% |
| Jun 2021 | 35% | 35% | 100% | 35% | 100% | 35% | 75% | 35% | 35% | 75% | 100% | 35% | 75% | 75% |

^a^ Site elected to do 100% of charts for this stratum

**Supplemental Table 2C. Overall sampling rates by site, surveillance period and age group ≥65 years, COVID-NET March 2020 – June 2021**

| **Surveillance Period** | **Sampling Rate by Site For 65+ Year Age Group** | | | | | | | | | | | | | |
| --- | --- | --- | --- | --- | --- | --- | --- | --- | --- | --- | --- | --- | --- | --- |
|  | **CA** | **CO** | **CT** | **GA** | **IA** | **MD** | **MI** | **MN** | **NM** | **NY** | **OH** | **OR** | **TN** | **UT** |
| Mar-May 2020 | 10% | 10% | 10% | 10% | 10% | 10% | 10% | 10%^a^ | 10% | 10% | 10% | 10% ^a^ | 10% ^a^ | 10% ^a^ |
| Jun-Sept 2020 | 10% | 10% | 10% | 5% | 10% | 5% | 10% | 10% ^a^ | 10% | 10% | 10% | 10% | 10% | 10% ^a^ |
| Oct 2020 | 13% | 3% | 16% | 5% | 38% | 2% | 11% | 4% | 7% | 24% | 9% | 11% | 5% | 13% |
| Nov 2020 | 5% | 3% | 5% | 3% | 5% | 3% | 5% | 5% | 5% | 5% | 5% | 5% | 5% | 5% ^a^ |
| Dec 2020 | 1% | 3% | 3% | 1% | 3% | 1% | 3% | 3% | 3% | 3% | 3% | 3% | 3% | 3% ^a^ |
| Jan 2021 | 1% | 5% | 5% | 1% | 5% | 1% | 5% | 5% | 5% | 5% | 5% | 5% | 5% | 5% |
| Feb 2021 | 3% | 7% | 7% | 3% | 50% | 3% | 30% | 7% | 7% | 7% | 30% | 30% | 7% | 30% |
| Mar 2021 | 10% | 10% | 10% | 3% | 50% | 3% | 10% | 10% | 10% | 10% | 30% | 30% | 10% | 30% |
| Apr2021 | 15% | 6% | 15% | 6% | 70% | 2% | 6% | 6% | 30% | 15% | 30% | 30% | 15% | 30% |
| May 2021 | 30% | 10% | 30% | 10% | 100% | 10% | 30% | 10% | 30% | 30% | 30% | 30% | 30% | 30% |
| Jun 2021 | 35% | 35% | 75% | 35% | 100% | 35% | 75% | 35% | 35% | 75% | 75% | 35% | 75% | 75% |

^a^ Site elected to do 100% of charts for this stratum

**Supplemental Table 3. Comparison of the distribution of demographic characteristics among all hospitalized cases versus weighted sampled hospitalized cases, COVID-NET, March 2020 – June 2021**

|  | **Mar 2020 – Jun 2021** | | **Mar 2020 – May 2020** | | **Jun 2020 – Sep 2020** | | **Oct 2020** | | **Nov 2020** | | **Dec 2020** | |  |
| --- | --- | --- | --- | --- | --- | --- | --- | --- | --- | --- | --- | --- | --- |
|  | All cases  Unweighted % | Sampled cases  Weighted % (95% CI) | All cases  Unweighted % | Sampled cases  Weighted % (95% CI) | All cases  Unweighted % | Sampled cases  Weighted % (95% CI) | All cases  Unweighted % | Sampled cases  Weighted % (95% CI) | All cases  Unweighted % | Sampled cases  Weighted % (95% CI) | All cases  Unweighted % | Sampled cases  Weighted % (95% CI) |  |
| **Age group (years) ^a^** |  |  |  |  |  |  |  |  |  |  |  |  |  |
| 18-49 | 26.7 | 26.7  (26.0-27.5) | 25.5 | 25.5  (24.2-26.8) | 33.5 | 33.5  (32.1-34.9) | 26.0 | 26.0  (23.4-28.7) | 22.1 | 22.1  (20.2-24.0) | 20.7 | 20.7  (18.0-23.6) |  |
| 50-64 | 27.9 | 27.9  (27.1-28.7) | 29.2 | 29.2  (27.7-30.7) | 27.8 | 27.8  (26.2-29.4) | 27.5 | 27.5  (24.7-30.3) | 26.3 | 26.3  (23.9-28.8) | 25.5 | 25.5  (23.2-28.0) |  |
| 65+ | 43.7 | 43.7  (42.5-44.9) | 44.4 | 44.4  (42.6-46.2) | 36.7 | 36.7  (34.5-38.9) | 44.9 | 44.9  (41.8-47.9) | 50.2 | 50.2  (47.4-53.0) | 52.4 | 52.4  (48.4-56.3) |  |
| **Sex** |  |  |  |  |  |  |  |  |  |  |  |  |  |
| Male | 50.2 | 50.2  (48.8-51.6) | 52.4 | 52.4  (50.8-53.9) | 49.2 | 49.2  (47.3-51.2) | 51.1 | 51.1  (47.0-55.2) | 51.2 | 51.2  (48.9-53.6) | 50.2 | 50.2  (45.1-55.3) |  |
| Female | 49.8 | 49.8  (48.4-51.2) | 47.6 | 47.6  (46.1-49.2) | 50.8 | 50.8  (48.8-52.7) | 48.9 | 48.9  (44.8-53.0) | 48.8 | 48.8  (46.4-51.1) | 49.8 | 49.8  (44.7-54.9) |  |
| **Race/Ethnicity ^b^** |  |  |  |  |  |  |  |  |  |  |  |  |  |
| NH White | 43.5 | 43.5  (42.2-44.8) | 34.3 | 34.3  (32.7-35.9) | 31.6 | 31.6  (29.3-34.0) | 46.4 | 46.4  (43.4-49.5) | 51.3 | 51.3  (48.7-53.9) | 50.2 | 50.2  (46.4-54.0) |  |
| NH Black | 27.2 | 27.2  (26.3-28.2) | 33.4 | 33.4  (31.7-35.2) | 31.9 | 31.9  (30.0-34.0) | 20.7 | 20.7  (18.3-23.3) | 18.9 | 18.9  (17.1-20.8) | 23.1 | 23.1  (20.7-25.6) |  |
| NH AI/AN | 1.3 | 1.4  (1.2-1.6) | 1.5 | 1.3  (1.1-1.7) | 1.1 | 1.2  (0.9-1.5) | 1.3 | 1.2  (0.7-2.1) | 1.7 | 2.5  (1.9-3.3) | 1.6 | 1.8  (1.2-2.7)  ) |  |
| NH Asian/PI | 4.9 | 5.7  (5.2-6.2) | 4.8 | 4.8  (4.3-5.5) | 5.8 | 6.3  (5.6-7.1) | 4.7 | 6.1  (4.8-7.7) | 4.8 | 5.3  (4.3-6.4) | 5.1 | 3.8  (2.3-5.8) |  |
| More than one race/ethnicity | 0.4 | 0.5  (0.4-0.7) | 0.3 | 0.3  (0.1-0.6) | 0.5 | 0.7  (0.4-1.1) | 0.7 | 0.7  (0.2-1.8) | 0.5 | 0.3  (0.1-0.7) | 0.4 | 0.8  (0.2-2.3) |  |
| Hispanic | 18.3 | 18.6  (17.5-19.7) | 22.5 | 23.2  (21.6-24.9) | 24.8 | 25.0  (23.3-26.8) | 22.6 | 21.2  (18.7-23.7) | 17.8 | 17.2  (15.2-19.4) | 15.2 | 17.1  (13.6-21.0) |  |
| **State** |  |  |  |  |  |  |  |  |  |  |  |  |  |
| CA | 7.4 | 7.4  (7.1-7.8) | 4.1 | 4.1  (3.6-4.7) | 11.1 | 11.1  (10.2-12.1) | 4.1 | 4.1  (3.0-5.5) | 4.3 | 4.3  (2.7-6.4) | 9.6 | 9.6  (8.1-11.4) |  |
| CO | 8.9 | 8.9  (8.5-9.4) | 12.2 | 12.2  (11.4-13.1) | 5.0 | 5.0  (4.2-6.0) | 12.6 | 12.6  (10.5-15.0) | 13.4 | 13.4  (11.9-15.1) | 7.6 | 7.6  (6.2-9.2) |  |
| CT | 5.0 | 5.0  (4.7-5.4) | 9.3 | 9.3  (8.6-10.2) | 1.5 | 1.5  (1.1-1.9) | 2.8 | 2.8  (1.9-4.0) | 5.1 | 5.1  (4.1-6.2) | 5.1 | 5.1  (4.0-6.4) |  |
| GA | 15.7 | 15.7  (15.2-16.2) | 11.8 | 11.8  (10.9-12.8) | 25.7 | 25.7  (24.4-27.0) | 12.4 | 12.4  (9.8-15.4) | 8.2 | 8.2  (7.0-9.6) | 13.1 | 13.1  (10.1-16.7) |  |
| IA | 0.5 | 0.9  (0.8-1.1) | 0.5 | 0.5  (0.3-0.7) | 0.6 | 0.4  (0.2-0.6) | 0.8 | 5.8  (4.4-7.3) | 0.9 | 0.7  (0.4-1.2) | 0.3 | 0.0  (0.0-0.3) |  |
| MD | 22.5 | 22.5  (21.3-23.8) | 32.4 | 32.4  (30.4-34.5) | 19.5 | 19.5  (16.8-22.3) | 16.7 | 16.7  (11.8-22.7) | 18.7 | 18.7  (16.9-20.5) | 19.8 | 19.8  (16.2-23.8) |  |
| MI | 3.8 | 3.8  (3.6-4.1) | 3.9 | 3.9  (3.5-4.5) | 1.0 | 1.0  (0.7-1.5) | 3.5 | 3.5  (2.5-4.8) | 4.5 | 4.5  (3.6-5.5) | 3.8 | 3.8  (2.9-5.0) |  |
| MN | 9.6 | 9.2  (8.8-9.6) | 8.5 | 8.5  (7.8-9.3) | 10.3 | 10.5  (9.6-11.4) | 14.3 | 9.3  (7.3-11.7) | 14.6 | 14.8  (13.1-16.6) | 8.5 | 8.9  (7.4-10.5) |  |
| NM | 4.3 | 4.3  (4.0-4.6) | 1.9 | 1.9  (1.6-2.3) | 3.3 | 3.3  (2.8-3.8) | 8.1 | 8.1  (6.5-9.9) | 7.2 | 7.2  (5.9-8.7) | 5.3 | 5.3  (3.3-7.9) |  |
| NY | 7.2 | 7.2  (6.8-7.6) | 4.9 | 4.9  (4.2-5.8) | 2.0 | 2.0  (1.6-2.5) | 3.3 | 3.3  (2.3-4.6) | 5.4 | 5.4  (4.4-6.6) | 11.8 | 11.8  (10.1-13.6) |  |
| OH | 2.6 | 2.6  (2.3-2.9) | 3.5 | 3.5  (2.9-4.3) | 4.5 | 4.5  (3.6-5.5) | 3.7 | 3.7  (2.7-5.1) | 3.1 | 3.1  (1.1-6.9) | 1.9 | 1.9  (1.2-2.8) |  |
| OR | 2.7 | 2.7  (2.2-3.2) | 1.6 | 1.6  (1.3-1.9) | 2.9 | 2.9  (2.3-3.6) | 2.8 | 2.8  (1.9-4.0) | 3.5 | 3.5  (2.7-4.4) | 2.7 | 2.7  (1.8-3.9) |  |
| TN | 6.6 | 6.6  (6.3-6.9) | 3.6 | 3.6  (3.2-4.2) | 8.5 | 8.5  (7.7-9.4) | 9.1 | 9.1  (7.4-11.2) | 7.2 | 7.2  (5.6-9.0) | 7.7 | 7.7  (6.3-9.3) |  |
| UT | 3.0 | 3.0  (2.7-3.3) | 1.6 | 1.6  (1.3-1.9) | 4.2 | 4.2  (3.3-5.2) | 5.7 | 5.7  (4.3-7.2) | 4.0 | 4.0  (3.1-5.0) | 2.7 | 2.7  (6.3-9.3) |  |

|  | **Jan 2021** | | **Feb 2021** | | **March 2021** | | **Apr 2021** | | **May 2021** | | **June 2021** | |  |
| --- | --- | --- | --- | --- | --- | --- | --- | --- | --- | --- | --- | --- | --- |
|  | All cases  Unweighted % | Sampled cases  Weighted % (95% CI) | All cases  Unweighted % | Sampled cases  Weighted % (95% CI) | All cases  Unweighted % | Sampled cases  Weighted % (95% CI) | All cases  Unweighted % | Sampled cases  Weighted % (95% CI) | All cases  Unweighted % | Sampled cases  Weighted % (95% CI) | All cases  Unweighted % | Sampled cases  Weighted % (95% CI) |  |
| **Age group (years)** |  |  |  |  |  |  |  |  |  |  |  |  |  |
| 18-49 | 21.3 | 21.3  (19.1-23.7) | 24.6 | 24.6  (21.2-28.3) | 29.6 | 29.6  (27.0-32.2) | 35.3 | 35.3  (32.7-37.9) | 37.6 | 37.6  (35.1-40.1) | 40.2 | 40.2  (37.1-43.5) |  |
| 50-64 | 26.3 | 26.3  (24.0-28.6) | 27.0 | 27.0  (24.3-29.9) | 32.0 | 32.0  (27.8-36.4) | 32.8 | 32.8  (30.0-35.6) | 29.8 | 29.7  (26.8-32.9) | 26.8 | 26.8  (24.3-29.4) |  |
| 65+ | 50.7 | 50.7  (47.5-53.9) | 46.2 | 46.2  (41.4-51.1) | 36.3 | 36.3  (30.8-42.1) | 29.7 | 29.7  (27.3-32.3)  ) | 29.4 | 29.5  (26.3-32.8) | 29.0 | 29.0  (26.3-32.0) |  |
| **Sex** |  |  |  |  |  |  |  |  |  |  |  |  |  |
| Male | 50.1 | 50.1  (43.3-56.9) | 50.6 | 50.6  (47.7-53.5) | 49.5 | 49.5  (44.2-54.7) | 48.7 | 48.7  (43.0-54.4) | 46.5 | 46.5  (41.1-52.0) | 47.5 | 47.5  (44.5-50.5) |  |
| Female | 49.9 | 49.9  (43.1-56.7) | 49.4 | 49.4  (46.5-52.3) | 50.5 | 50.5  (45.3-55.8) | 51.3 | 51.3  (45.6-57.0) | 53.5 | 53.5  (48.0-58.9) | 52.5 | 52.5  (49.5-55.5) |  |
| **Race/Ethnicity ^b^** |  |  |  |  |  |  |  |  |  |  |  |  |  |
| NH White | 48.0 | 48.0  (43.5-52.6) | 45.5 | 45.5  (38.7-52.4) | 47.4 | 47.4  (44.5-50.2) | 46.7 | 46.7  (39.4-54.1) | 45.6 | 45.7  (42.2-49.1) | 45.9 | 46.0  (43.1-48.8) |  |
| NH Black | 24.9 | 24.9  (21.6-28.5) | 27.9 | 27.9  (23.9-32.1) | 30.5 | 30.5  (27.8-33.2) | 31.6 | 31.5  (22.3-42.1) | 28.8 | 28.7  (24.8-32.9) | 25.2 | 25.1  (22.4-28.0) |  |
| NH AI/AN | 1.5 | 1.4  (0.6-2.7) | 1.0 | 1.0  (0.4-2.0) | 0.6 | 0.7  (0.2-2.0) | 0.7 | 0.4  (0.1-0.9) | 1.7 | 1.2  (0.7-1.9) | 1.5 | 1.2  (0.6-2.0) |  |
| NH Asian/PI | 5.4 | 8.3  (5.1-12.6) | 5.2 | 7.2  (5.7-9.1) | 3.9 | 5.7  (4.4-7.3) | 3.9 | 6.5  (4.0-9.8) | 3.1 | 2.7  (1.9-3.6) | 4.0 | 4.4  (3.3-5.7) |  |
| More than one race/ethnicity | 0.4 | 0.6  (0.1-1.7) | 0.4 | 0.2  (0.0-0.6) | 0.4 | 0.4  (0.1-0.9) | 0.3 | 0.3  (0.0-1.0) | 0.7 | 0.9  (0.2-2.4) | 0.7 | 1.0  (0.4-2.0) |  |
| Hispanic | 15.1 | 14.5  (11.1-18.4) | 15.2 | 14.5  (11.5-18.0) | 12.7 | 13.2  (10.2-16.7) | 12.5 | 12.8  (11.0-14.7) | 15.9 | 17.9  (15.1-20.9) | 18.5 | 19.3  (17.1-21.7) |  |
| **State** |  |  |  |  |  |  |  |  |  |  |  |  |  |
| CA | 11.3 | 11.3  (9.7-13.1) | 9.6 | 9.6  (8.0-11.4) | 5.4 | 5.4  (4.2-6.8) | 3.4 | 3.4  (2.5-4.6) | 4.6 | 4.6  (3.6-5.8) | 9.4 | 9.4  (7.7-11.2) |  |
| CO | 5.3 | 5.3  (4.2-6.6) | 6.9 | 6.9  (4.1-10.9) | 7.0 | 7.0  (5.6-8.5) | 9.3 | 9.3  (6.8-12.3) | 13.7 | 13.7  (12.0-15.5) | 12.3 | 12.2  (10.4-14.2) |  |
| CT | 4.4 | 4.4  (3.1-6.2) | 5.8 | 5.8  (4.5-7.2) | 7.2 | 7.2  (5.8-8.8) | 5.3 | 5.3  (4.2-6.7) | 3.3 | 3.3  (2.5-4.3) | 3.1 | 3.1  (2.2-4.2) |  |
| GA | 19.9 | 19.9  (17.8-22.0) | 20.4 | 20.4  (16.2-25.1) | 15.7 | 15.7  (13.2-18.5) | 12.1 | 12.1  (8.8-16.2) | 13.4 | 13.4  (11.7-15.2) | 16.0 | 16.0  (13.8-18.4) |  |
| IA | 0.3 | 0.6  (0.3-1.1) | 0.3 | 1.6  (1.0-2.5) | 0.2 | 0.8  (0.4-1.4) | 0.1 | 1.3  (0.8-2.1) | 0.1 | 1.4  (0.9-2.1) | 1.1 | 2.8  (1.5-4.7) |  |
| MD | 21.5 | 21.5  (17.9-25.5) | 23.2 | 23.2  (17.8-29.3) | 26.5 | 26.5  (24.1-29.1) | 26.9 | 26.9  (22.6-31.5) | 19.4 | 19.4  (17.5-21.5) | 12.7 | 12.7  (10.4-15.2) |  |
| MI | 2.3 | 2.3  (1.6-3.3) | 2.1 | 2.1  (1.4-3.1) | 6.4 | 6.4  (5.1-7.9) | 10.1 | 10.1  (8.3-12.0) | 5.7 | 5.7  (4.6-7.0) | 2.9 | 2.9  (2.0-4.0) |  |
| MN | 4.7 | 4.5  (3.3-6.0) | 6.3 | 5.0  (3.9-6.4) | 9.0 | 8.4  (6.9-10.1) | 12.2 | 11.0  (8.7-13.7) | 11.6 | 10.4  (8.9-12.0) | 10.4 | 8.7  (7.1-10.4) |  |
| NM | 4.8 | 4.8  (3.8-6.0) | 4.0 | 4.0  (2.9-5.3) | 2.7 | 2.7  (1.8-3.9) | 2.4 | 2.4  (1.6-3.4) | 4.6 | 4.6  (3.6-5.7) | 6.3 | 6.3  (5.0-7.8) |  |
| NY | 12.1 | 12.1  (10.5-13.9) | 9.1 | 9.1  (7.0-11.4) | 8.0 | 8.0  (6.6-9.7) | 8.0 | 8.0  (6.4-9.8) | 9.7 | 9.7  (8.3-11.4) | 6.4 | 6.4  (5.1-8.0) |  |
| OH | 1.3 | 1.3  (0.8-2.1) | 1.2 | 1.2  (0.7-2.0) | 1.3 | 1.3  (0.8-2.1) | 1.3 | 1.3  (0.8-2.1) | 1.7 | 1.7  (0.9-2.9) | 1.7 | 1.7  (1.1-2.6) |  |
| OR | 2.1 | 2.1  (0.5-5.7) | 2.0 | 2.0  (1.3-3.0) | 1.8 | 1.8  (1.2-2.8) | 2.9 | 2.9  (2.1-4.0) | 5.4 | 5.4  (4.3-6.7) | 7.2 | 7.2  (5.8-8.9) |  |
| TN | 7.0 | 7.0  (5.8-8.5) | 6.4 | 6.4  (5.1-8.0) | 6.6 | 6.6  (5.1-8.5) | 4.6 | 4.6  (3.5-5.8) | 4.0 | 4.0  (3.1-5.1) | 4.2 | 4.2  (3.1-5.5) |  |
| UT | 2.8 | 2.8  (1.6-4.5) | 2.7 | 2.7  (1.8-3.7) | 2.1 | 2.1  (1.3-3.0) | 1.4 | 1.4  (0.8-2.2) | 2.7 | 2.7  (1.9-3.6) | 6.5 | 6.5  (5.1-8.0) |  |

^a^ Percentages do not add up to 100% because pediatric cases 0-17 years, who were sampled at a rate of 100%, are not shown.

^b^ NH= Non-Hispanic; AI/AN= American Indian and Alaska Native; Asian/PI= Asian or Pacific Islander; Hispanic= Hispanic or Latino; Race/ethnicity distributions do not add to 100% due to 3-5% of cases missing data on race and ethnicity.

**Supplemental Table 4. Weighted percentages and confidence intervals for select clinical interventions and outcomes by month and age group among sampled hospitalized cases, COVID-NET^a^, March 2020 – June 2021**

| **Clinical Interventions/Outcomes** | **18-49 years** | **50-64 years** | **65+ years** | **Overall** |
| --- | --- | --- | --- | --- |
|  | % (95% CI) | % (95% CI) | % (95% CI) | % (95% CI) |
| **March 2020** |  |  |  |  |
| ICU Admission | 29.0 (22.3-36.5) | 38.4 (31.8-45.3) | 42.3 (35.4-49.4) | 37.8 (34.7-40.9) |
| Mechanical Ventilation | 18.9 (13.5-25.3) | 27.2 (21.4-33.7) | 32.9 (26.5-39.7) | 27.5 (24.7-30.4) |
| In-hospital death | 2.7 (1.0-5.7) ^b^ | 9.3 (3.7-18.6) ^b^ | 23.8 (17.6-30.9) | 13.8 (10.4-17.9) |
| **April 2020** |  |  |  |  |
| ICU Admission | 28.4 (25.4-31.6) | 36.3 (32.2-40.6) | 34.2 (30.6-38.0) | 33.5 (31.7-35.4) |
| Mechanical Ventilation | 15.6 (13.6-17.7) | 24.2 (19.8-29.0) | 21.2 (18.1-24.6) | 20.7 (19.1-22.4) |
| In-hospital death | 3.7 (2.8-4.9) | 11.2 (8.7-14.3) | 26.0 (22.7-29.6) | 16.5 (14.9-18.2) |
| **May 2020** |  |  |  |  |
| ICU Admission | 20.2 (17.2-23.6) | 33.3 (26.1-41.1) | 30.9 (26.3-35.7) | 28.4 (25.8-31.2) |
| Mechanical Ventilation | 9.3 (7.6-11.2) | 21.9 (15.5-29.4) | 18.5 (14.7-22.7) | 16.5 (14.4-18.9) |
| In-hospital death | 1.7 (0.4-4.9) ^b^ | 10.3 (7.4-13.8) | 23.9 (19.7-28.4) | 13.3 (11.7-14.9) |
| **June 2020** |  |  |  |  |
| ICU Admission | 16.1 (11.6-21.4) | 28.3 (22.5-34.6) | 41.0 (32.7-49.6) | 28.1 (25.2-31.1) |
| Mechanical Ventilation | 4.3 (2.0-7.9) ^b^ | 15.7 (10.4-22.3) | 16.9 (11.1-24.2) | 11.7 (9.7-14.0) |
| In-hospital death | 2.0 (0.3-6.3) ^b^ | 6.7 (3.8-10.8) | 13.2 (8.1-19.9) | 7.1 (5.2-9.3) |
| **July 2020** |  |  |  |  |
| ICU Admission | 18.5 (15.7-21.7) | 28.6 (23.2-34.5) | 32.5 (24.6-41.2) | 26.6 (24.4-28.9) |
| Mechanical Ventilation | 7.0 (5.1-9.3) | 12.4 (8.2-17.8) | 20.1 (15.5-25.4) | 13.2 (10.6-16.1) |
| In-hospital death | 1.7 (0.2-6.5) ^b^ | 6.9 (4.5-10.1) | 20.9 (14.5-28.5) | 10.0 (6.7-14.2) |
| **August 2020** |  |  |  |  |
| ICU Admission | 17.8 (14.5-21.6) | 24.3 (18.8-30.4) | 21.8 (16.8-27.4) | 21.3 (19.0-23.8) |
| Mechanical Ventilation | 6.7 (3.6-11.2) | 10.6 (6.9-15.3) | 12.2 (8.0-17.6) | 9.8 (8.2-11.7) |
| In-hospital death | 1.0 (0.2-2.8) ^b^ | 4.5 (0.8-13.5) ^b^ | 17.2 (12.7-22.5) | 8.0 (6.3-9.9) |
| **September 2020** |  |  |  |  |
| ICU Admission | 16.1 (12.5-20.2) | 27.1 (19.2-36.2) | 25.4 (18.8-33.0) | 23.2 (20.1-26.4) |
| Mechanical Ventilation | 8.0 (3.8-14.6) ^b^ | 14.1 (9.2-20.3) | 11.5 (5.2-21.2) ^b^ | 11.1 (7.0-16.5) |
| In-hospital death | 2.8 (1.4-5.0) | 5.2 (2.6-9.2) | 19.2 (11.3-29.5) | 10.1 (6.2-15.3) |
| **October 2020** |  |  |  |  |
| ICU Admission | 13.5 (8.6-19.9) | 23.0 (13.7-34.7)^c^ | 24.8 (20.1-30.1) | 21.2 (18.8-23.8) |
| Mechanical Ventilation | 5.3 (2.7-9.2) | 9.6 (5.2-15.9) | 9.7 (6.6-13.6) | 8.4 (6.8-10.3) |
| In-hospital death | 2.0 (0.7-4.4) ^b^ | 6.7 (2.7-13.5) ^b^ | 13.2 (9.7-17.6) | 8.3 (6.3-10.7) |
| **November 2020** |  |  |  |  |
| ICU Admission | 17.4 (13.8-21.5) | 22.7 (18.1-27.9) | 22.1 (15.4-30.1) | 21.3 (17.0-26.1) |
| Mechanical Ventilation | 10.2 (7.5-13.4) | 11.8 (8.8-15.5) | 10.4 (5.6-17.2) | 10.7 (7.4-14.7) |
| In-hospital death | 2.1 (0.9-4.2) ^b^ | 6.7 (4.5-9.5) | 16.0 (11.4-21.4) | 10.2 (7.7-13.2) |
| **December 2020** |  |  |  |  |
| ICU Admission | 17.8 (11.8-25.3) | 20.7 (16.2-25.8) | 21.7 (16.9-27.2) | 20.7 (17.6-24.1) |
| Mechanical Ventilation | 9.9 (6.5-14.2) | 12.5 (8.9-16.8) | 15.4 (10.4-21.6) | 13.4 (10.7-16.5) |
| In-hospital death | 2.0 (0.7-4.5) | 9.2 (6.2-13.1) | 19.5 (13.5-26.8) | 13.0 (9.6-17.2) |
| **January 2021** |  |  |  |  |
| ICU Admission | 15.9 (9.5-24.3) | 24.9 (20.7-29.6) | 22.9 (18.7-27.6) | 22.0 (19.6-24.6) |
| Mechanical Ventilation | 8.8 (4.8-14.4) | 14.9 (10.9-19.7) | 14.5 (11.0-18.5) | 13.2 (11.1-15.6) |
| In-hospital death | 4.2 (2.2-7.2) | 10.5 (7.6-14.0) | 14.5 (9.6-20.5) | 11.0 (8.3-14.2) |
| **February 2021** |  |  |  |  |
| ICU Admission | 12.9 (9.5-17.0) | 19.3 (15.1-24.1) | 16.9 (8.8-28.0) | 16.8 (12.9-21.4) |
| Mechanical Ventilation | 8.8 (5.6-13.2) | 11.0 (7.1-16.0) | 8.3 (5.6-11.8) | 9.1 (7.5-11.0) |
| In-hospital death | 1.2 (0.1-5.2) ^b^ | 6.3 (3.9-9.6) | 9.0 (6.1-12.5) | 6.2 (4.6-8.0) |
| **March 2021** |  |  |  |  |
| ICU Admission | 13.7 (10.3-17.8) | 18.3 (14.4-22.8) | 19.7 (12.1-29.3) | 17.6 (14.8-20.7) |
| Mechanical Ventilation | 8.6 (5.5-12.6) | 9.1 (6.1-12.8) | 12.2 (7.7-18.1) | 10.1 (7.2-13.6) |
| In-hospital death | 1.8 (0.7-4.0) ^b^ | 6.5 (4.1-9.6) | 11.2 (5.7-19.3) | 6.7 (4.3-9.8) |
| **April 2021** |  |  |  |  |
| ICU Admission | 14.9 (11.2-19.3) | 18.5 (12.6-25.8) | 20.2 (15.3-25.9) | 17.9 (15.6-20.4) |
| Mechanical Ventilation | 10.0 (6.9-13.8) | 10.2 (6.3-15.5) | 9.9 (6.9-13.6) | 9.9 (8.4-11.7) |
| In-hospital death | 3.3 (1.2-7.3) ^b^ | 6.1 (3.6-9.5) | 11.2 (7.8-15.5) | 6.5 (5.1-8.2) |
| **May 2021** |  |  |  |  |
| ICU Admission | 13.7 (10.8-17.1) | 23.1 (18.7-28.0) | 22.0 (17.9-26.5) | 19.4 (17.4-21.5) |
| Mechanical Ventilation | 5.6 (3.5-8.3) | 9.9 (6.9-13.8) | 10.2 (6.7-14.8) | 8.3 (6.5-10.4) |
| In-hospital death | 2.0 (0.7-4.3) ^b^ | 5.6 (3.3-8.8) | 12.3 (9.3-15.8) | 6.1 (4.4-8.2) |
| **June 2021** |  |  |  |  |
| ICU Admission | 13.6 (10.2-17.5) | 22.3 (17.9-27.1) | 22.9 (17.8-28.8) | 18.8 (16.6-21.1) |
| Mechanical Ventilation | 6.4 (3.9-9.8) | 12.5 (9.2-16.5) | 10.3 (6.3-15.6) | 9.3 (7.3-11.6) |
| In-hospital death | 2.0 (0.7-4.2) ^b^ | 5.9 (3.6-8.9) | 10.4 (7.4-14.2) | 5.4 (3.8-7.3) |

ICU = Intensive Care Unit;

^a^ COVID-NET includes 14 U.S. states (CA, CO, CT, IA, GA, MD, MI, MN, NM, NY, OH, OR, TN, UT)

^b^ Relative standard error (RSE) > 30

^c^ Confidence interval half width > 10

**Supplemental Table 5. COVID-NET weighting summary by surveillance period and age group, March 2020 – June 2021**

| **Month** | **18-49 years** | | | | **50-64 years** | | | | **65+ years** | | | |
| --- | --- | --- | --- | --- | --- | --- | --- | --- | --- | --- | --- | --- |
|  | **Total cases** | **Sampled cases** | **Overall weight (range)** | **WEFF** | **Total cases** | **Sampled cases** | **Overall weight (range)** | **WEFF** | **Total cases** | **Sampled cases** | **Overall weight (range)** | **WEFF** |
| Mar 2020 | 1252 | 540 | 2.3 (0.8-11.3) | 2.5 | 1688 | 213 | 7.9 (3.4-16.5) | 1.2 | 2182 | 207 | 10.5 (4.3-15.6) | 1.0 |
| Apr 2020 | 3051 | 1309 | 2.3 (0.9-10.1) | 2.6 | 3819 | 522 | 7.3 (2.5-16.0) | 1.2 | 6305 | 648 | 9.7 (2.5-14.6) | 1.0 |
| May 2020 | 3087 | 1412 | 2.2 (0.8-12.6) | 2.6 | 2952 | 388 | 7.6 (2.3-15.8) | 1.3 | 4380 | 392 | 11.2 (2.4-15.1) | 1.1 |
| Jun 2020 | 2255 | 450 | 5.0 (1.0-28.9) | 3.0 | 1731 | 230 | 7.5 (3.0-25.2) | 1.8 | 2143 | 143 | 15.0 (8.9-31.7) | 1.3 |
| Jul 2020 | 3674 | 701 | 5.2 (1-24.3) | 2.6 | 3040 | 359 | 8.5 (3.4-23.7) | 1.5 | 3830 | 273 | 14.0 (7.1-26.7) | 1.1 |
| Aug 2020 | 2517 | 515 | 4.9 (1-25.5) | 2.5 | 2112 | 227 | 9.3 (3.6-27.0) | 1.5 | 2846 | 249 | 11.4 (4.4-23.9) | 1.2 |
| Sept 2020 | 1690 | 386 | 4.4 (1-23.1) | 2.6 | 1530 | 206 | 7.4(3.5-22.6) | 1.5 | 2290 | 173 | 13.2 (5.6-23.5) | 1.1 |
| Oct 2020 | 2532 | 275 | 7.1 (1.9-30.4) | 1.6 | 2677 | 312 | 8.6 (1.7-32.8) | 1.7 | 4373 | 311 | 14.1 (3.8-44.0) | 1.4 |
| Nov 2020 | 4965 | 453 | 11.0 (7.1-20.3) | 1.1 | 5918 | 541 | 10.9 (7.8-19.4) | 1.1 | 11294 | 503 | 22.5 (17.0-48.0) | 1.1 |
| Dec 2020 | 5778 | 263 | 22.0 (3.2-47.9) | 1.3 | 7125 | 295 | 24.2 (3.8-47.0) | 1.1 | 14615 | 362 | 40.4 (10.8-124.8) | 1.4 |
| Jan 2021 | 5227 | 289 | 18.1 (4.9-62.8) | 1.7 | 6441 | 380 | 17.0 (5.5-61.5) | 1.9 | 12429 | 366 | 34.0 (16.0-121.8) | 1.6 |
| Feb 2021 | 2760 | 332 | 8.3 (2.7-22.4) | 1.3 | 3030 | 317 | 9.6 (2.7-18.4) | 1.3 | 5183 | 340 | 15.2 (2.9-40.5) | 1.5 |
| Mar 2021 | 3057 | 377 | 8.1 (2.5-19.5) | 1.3 | 3302 | 351 | 9.4 (2.3-46.8) | 2.1 | 3749 | 282 | 13.3 (3.4-54.0) | 1.9 |
| Apr 2021 | 4697 | 325 | 14.5 (2.7-63.7) | 1.8 | 4362 | 353 | 12.4 (2.4-59.1) | 2.0 | 3961 | 338 | 11.7 (2.6-45.0) | 1.8 |
| May 2021 | 2868 | 478 | 6.0 (2.5-16.5) | 1.7 | 2270 | 391 | 5.8 (2.6-13.9) | 1.3 | 2249 | 422 | 5.3 (2.5-12.0) | 1.3 |
| Jun 2021 | 1178 | 365 | 3.2 (1-6.1) | 1.2 | 785 | 342 | 3.9 (1-2.3) | 1.1 | 850 | 387 | 2.2 (1.1-4.5) | 1.2 |

Abbreviation: WEFF = Weighting Effect
